# The Double-*K* Fracture Toughness of Concrete with Different Coarse Aggregate Volume Fractions

**DOI:** 10.3390/ma18245526

**Published:** 2025-12-09

**Authors:** Xiao Li, Ying Zhang, Yanwei Chen, Ying Yuan, Jili Feng, Zhiguang Li

**Affiliations:** 1 School of Civil and Architectural Engineering, Hebei University of Engineering Science, Shijiazhuang 050091, China; lixiao2025@yeah.net; 2 Hebei Technology Innovation Center for Intelligent Development and Control of Underground Built Environment, Hebei GEO University, Shijiazhuang 052161, China; luck_08zhangying@163.com (Y.Z.); yuanyingson@163.com (Y.Y.); lizhg1978@126.com (Z.L.); 3 State Key Laboratory for Tunnel Engineering, China University of Mining and Technology, Beijing 100083, China; fjl@cumtb.edu.cn

**Keywords:** aggregate proportion, crack propagation, fracture properties, wedge-splitting test, safety warning parameter

## Abstract

This study examines how coarse aggregate volume fraction (*V*_a_) affects the double-*K* fracture toughness and fundamental mechanical properties of concrete. Wedge-splitting tests were conducted on specimens with six different *V*_a_ values: 19%, 25%, 31%, 37%, 43%, and 50%. The results indicate that compressive strength (*f*_c_) and elastic modulus (*E*) consistently increase with *V*_a_, reaching 59.8 MPa and 37.9 GPa at *V*_a_ = 50%, respectively. Conversely, tensile strength (*f*_t_), double-*K* fracture toughness (including initiation toughness $K_{\mathrm{IC}}^{\mathrm{ini}}$ and unstable toughness $K_{\mathrm{IC}}^{\mathrm{un}}$), and fracture energy (*G*_IF_) initially increase before decreasing, peaking at an optimal *V*_a_ of 37%. Specifically, $K_{\mathrm{IC}}^{\mathrm{ini}}$, $K_{\mathrm{IC}}^{\mathrm{un}}$, and *G*_IF_ reached their maximum values of 0.54 MPa·m^1/2^, 1.20 MPa·m^1/2^, and 225.0 N/m at *V*_a_ = 37%. The tortuosity of crack paths follows a similar trend, becoming more pronounced up to *V*_a_ = 37% before diminishing. Furthermore, quantitative exponential relationships were established between *f*_t_ and $K_{\mathrm{IC}}^{\mathrm{ini}}$, $K_{\mathrm{IC}}^{\mathrm{un}}$, and *G*_IF_. A safety warning parameter (*δ*), derived from the double-*K* fracture toughness, was proposed to quantitatively assess the pre-peak ductility, with values ranging from 0.88 to 0.72 in this study. The findings offer valuable guidance for optimizing concrete mix design, suggesting that a *V*_a_ range of 25% to 31% provides an optimal balance between high crack initiation resistance and adequate safety warning capacity for critical engineering structures.

## 1. Introduction

As the most widely used construction material worldwide, the safety and durability of concrete are crucial for the stability of infrastructure. Concrete is a typical quasi-brittle material, and its entire life cycle is invariably accompanied by the initiation and propagation of cracks, such as pores induced during vibration, shrinkage cracks during hardening, thermal-induced cracks, and crack development under loading [1,2]. This progressive deterioration does not occur instantaneously but evolves through a complex process involving the nucleation and coalescence of micro-cracks, ultimately leading to macro-crack propagation and structural failure [3]. Consequently, the accurate characterization of these behaviors using appropriate fracture mechanics theories is of critical importance for concrete mix design and structural safety [4].

For multiphase composite materials like concrete, the plastic zone at the crack tip is referred to as the Fracture Process Zone (FPZ) [5]. Given the considerable dimensions of the FPZ, Shah and McGarry [6] argued that the principles of Linear Elastic Fracture Mechanics (LEFM) have limited applicability to concrete. Therefore, it is crucial to develop nonlinear fracture models for concrete that account for its unique characteristics and accurately describe its fracture behavior. Nonlinear fracture models for quasi-brittle materials often treat the complex FPZ either as a fictitious crack or an equivalent elastic crack [7]. Fictitious crack models, such as Hillerborg’s fictitious crack model [8] and Bazant’s crack band model [9], are typically used for numerical simulations. In contrast, the equivalent elastic crack models idealize the FPZ as an elastic crack, offering high computational efficiency and thus being commonly used for analytical solutions. Typical models based on this method include the two-parameter model (TPM) [10], the size effect model (SEM) [11], and the double-*K* fracture model (DKFM) [12,13,14].

Substantial experimental research has demonstrated that the fracture process of concrete can be divided into three distinct stages: crack initiation, stable propagation, and unstable fracture [12,13,14,15,16,17]. While conventional concrete structures typically focus only on the unstable fracture, critical engineering structures such as high concrete dams and nuclear containment shells require accurate prediction of crack initiation. To characterize this complete fracture process, Xu and Reinhardt [12,13,14] proposed the double-*K* fracture model (DKFM), defining two key parameters: initiation toughness ($K_{\mathrm{IC}}^{\mathrm{ini}}$) and unstable toughness ($K_{\mathrm{IC}}^{\mathrm{un}}$), corresponding to the critical states of crack initiation and unstable fracture, respectively. Based on the relationship between the stress intensity factors at different stages and double-*K* fracture toughness ($K_{\mathrm{IC}}^{\mathrm{ini}}$ and $K_{\mathrm{IC}}^{\mathrm{un}}$), a comprehensive double-*K* fracture criterion was subsequently established [18,19,20].

Within the framework of DKFM, extensive experimental investigations have been conducted on concrete fracture. For instance, Zhang et al. [17] analyzed the advantages and limitations of applying DKFM to cementitious composites. Kucharczyková et al. [21] utilized DKFM to evaluate the freeze–thaw resistance of concrete. Furthermore, recent studies have increasingly focused on the influence of modern materials incorporated into mixtures—such as steel-slag powder [22], basalt fiber [23], calcium aluminate cement [24], volcanic scoria coarse aggregate [25], as well as waste tire rubber and silica fume [26]—on $K_{\mathrm{IC}}^{\mathrm{ini}}$ and $K_{\mathrm{IC}}^{\mathrm{un}}$ of concrete.

Coarse aggregate, which is granular material with a particle size greater than 4.75 mm [27], occupies a substantial volume fraction in concrete and plays a critical role in influencing its fracture properties through parameters such as particle size, type, and content. Experimental data indicate that increasing the coarse aggregate size within a certain range effectively enhances the fracture energy (*G*_IF_) [28,29]. Furthermore, the mechanical properties (e.g., hardness, strength) and surface characteristics (e.g., roughness, shape) of coarse aggregates significantly influence the fracture parameters. For instance, under the same mix proportion, concrete prepared with gravel coarse aggregates exhibits notably higher fracture energy (*G*_IF_) and fracture toughness (*K*_IC_) compared to that made with limestone coarse aggregates [30,31]. Given its substantial magnitude (reaching up to approximately 70%), the coarse aggregate volume fraction exerts a particularly pronounced influence on the fracture behavior. Substantial experimental evidence confirms that within a reasonable range (approximately 15–40%), increasing the coarse aggregate volume fraction leads to significant improvements in *K*_IC_ and *G*_IF_ of self-compacting concrete [32,33], normal concrete [32], and high-strength concrete [34]. A similar toughening trend has also been observed for Mode II fracture toughness [35]. However, it is noteworthy that for high-strength concrete with a low water-to-binder ratio, further increasing the volume fraction to about 40–60% results in a decline in *K*_IC_ and *G*_IF_ [34,36], indicating a deterioration in fracture performance. It is worth pointing out that existing studies have primarily concentrated on the influence of coarse aggregate volume fraction on fracture energy (*G*_IF_) and fracture toughness (*K*_IC_), while research on its correlation with double-*K* fracture parameters ($K_{\mathrm{IC}}^{\mathrm{ini}}$ and $K_{\mathrm{IC}}^{\mathrm{un}}$) remains relatively limited.

This study aims to systematically investigate the effect of the coarse aggregate volume fraction on the complete fracture behavior of concrete, with a specific focus on the double-*K* fracture parameters. To this end, wedge-splitting tests were conducted on concrete specimens with six different coarse aggregate volume fractions. The objectives are (1) to determine the variations in fundamental mechanical properties (compressive strength *f*_c_, tensile strength *f*_t_, elastic modulus *E*) and key fracture parameters (initiation toughness $K_{\mathrm{IC}}^{\mathrm{ini}}$, unstable fracture toughness $K_{\mathrm{IC}}^{\mathrm{un}}$, fracture energy *G*_IF_, characteristic length *l*_ch_) with increasing aggregate content; (2) to analyze the resulting crack paths and fracture surfaces; (3) to establish quantitative relationships between tensile strength and the fracture parameters; and (4) to propose a novel safety warning parameter for quantitatively assessing the pre-peak ductility and early warning capacity of concrete structures.

## 2. Materials and Methods

This experimental investigation was conducted to systematically elucidate the influence of coarse aggregate volume fraction on the double-*K* fracture behavior of concrete. This research was carried out sequentially through four major stages, encompassing material preparation, mechanical testing, fracture parameter determination, and data analysis.

The major stages of this investigation are as follows:

Material Preparation and Specimen Casting: Design and preparation of concrete mixtures with six distinct coarse aggregate volume fractions. Casting and curing of wedge-splitting specimens and specimens for fundamental property tests.

Basic Mechanical Characterization: Experimental determination of the fundamental mechanical properties, including compressive strength, splitting tensile strength, and elastic modulus.

Fracture Testing and Phenomenological Analysis: Conducting wedge-splitting tests to obtain the load–crack mouth opening displacement (*P*-CMOD) curves. Simultaneously, the crack propagation process and final fracture patterns were recorded and analyzed.

Data Processing and Parametric Analysis: Calculating the double-*K* fracture parameters and characteristic length from the experimental data. Establishing quantitative relationships between the parameters and proposing a safety warning parameter.

The subsequent sections provide a detailed description of the materials, mix proportions, and experimental methods employed in these stages.

### 2.1. Specimen Preparation

**Figure 1:**
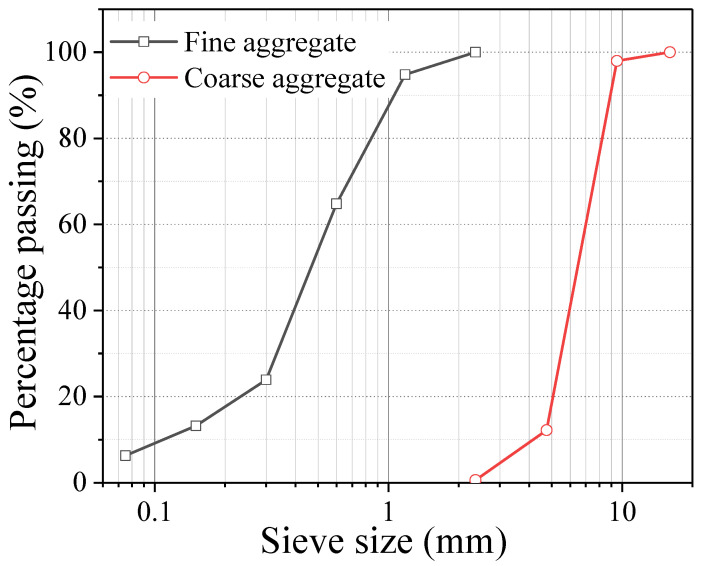
Particle size distribution of fine and coarse aggregates [15,35].

The raw materials used in the experiment to produce concrete included cement, coarse aggregate, fine aggregate, supplementary cementitious materials, water, and a water reducer. Portland cement P·O 42.5, produced by Beijing Jinyu Cement Factory in China, with a density of 3150 kg/m^3^ and a specific surface area of 329 m^2^/kg, was employed. The chemical and phase compositions of the cement, as provided by the manufacturer in the factory test report and determined according to Chinese National Standards GB/T 176-2017 [37] (for chemical analysis) and GB/T 40407-2021 [38] (for phase composition calculation), are presented in Table 1. The physical properties of the cement (test methods according to Chinese National Standards GB/T 8074-2008 [39] for specific surface area, GB/T 17671-2021 [40] for compressive and tensile strength, and GB/T 1346-2011 [41] for setting time) are summarized in Table 2. The coarse aggregate from Mentougou District, Beijing, China, consisted of continuously graded 5–16 mm limestone crushed stone [27], exhibiting an apparent density of 2690 kg/m^3^ and a bulk density of 1480 kg/m^3^. Natural river sand, from Mentougou District, Beijing, China, with a fineness modulus of 2.34, falling within the medium sand range, served as the fine aggregate. The particle size distributions of aggregates are shown in Figure 1. Municipal tap water from Beijing was used for mixing. To enhance concrete workability, a JFL-5 naphthalene-based high-range water reducer was incorporated. Furthermore, ultrafine limestone powder (specific gravity: 2.69, primary component: CaCO_3_) from Hezhou, Guangxi, was added to reduce cement content, optimize microstructure, and improve mechanical properties.

**Figure 2:**
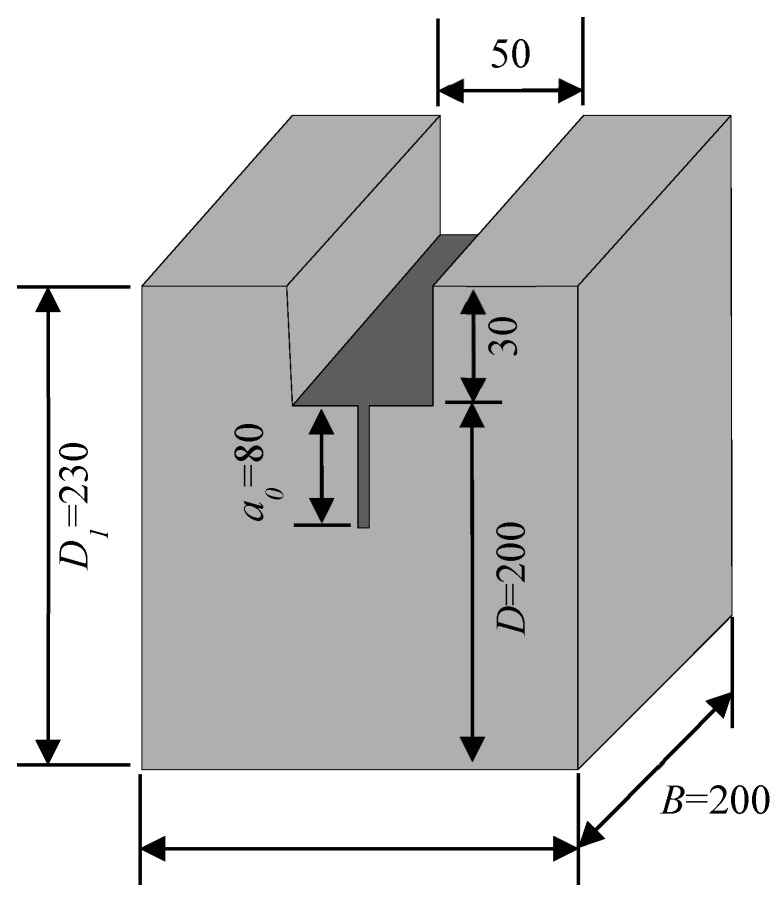
Geometries of the wedge-splitting specimen (mm) [15].

**Figure 3:**
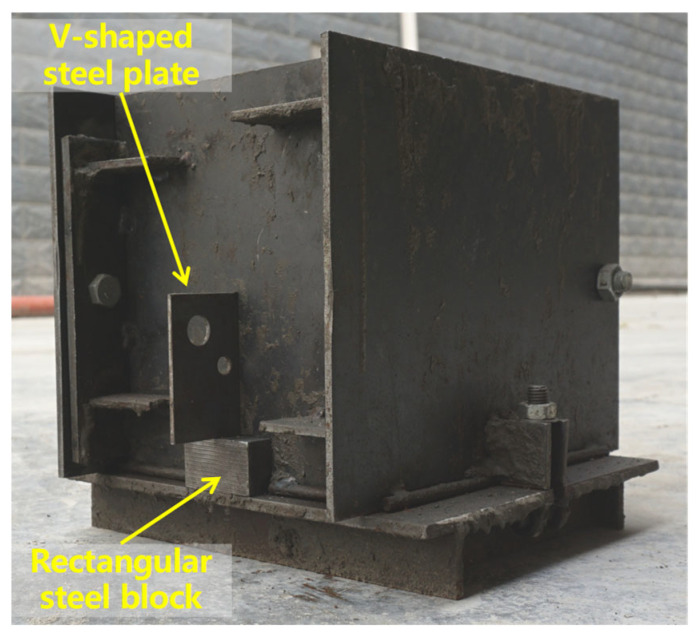
Mold composition of concrete specimens.

**Figure 4:**
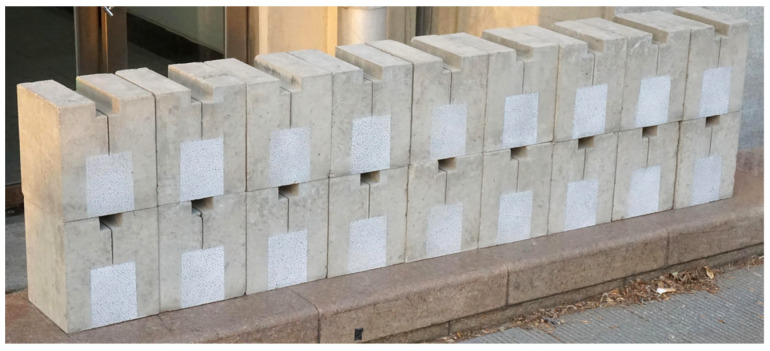
The final appearance of the prepared specimens.

The mix design for this experimental program was based on the principle of maintaining a constant total mass of coarse and fine aggregates. We established six distinct levels of coarse aggregate volume fraction, ranging from 19% to 50%. Detailed mix proportions are provided in Table 3. For each mix proportion, five wedge-splitting test (WST) specimens were prepared, with the geometric dimensions illustrated in Figure 2. WST specimens were utilized to determine several fracture parameters, including initiation toughness ($K_{\mathrm{IC}}^{\mathrm{ini}}$), unstable toughness ($K_{\mathrm{IC}}^{\mathrm{un}}$), fracture energy (*G*_IF_), and characteristic length (*l*_ch_). To ensure the stability of the top groove and pre-formed notch geometry, an inverse casting sequence was adopted. Before casting, a rectangular steel block and a V-shaped steel plate were pre-positioned at the mold bottom to simultaneously form the groove and notch (see Figure 3). The specimens were demolded one day after casting and cured for 28 days under standard laboratory conditions (a temperature of 20 ± 2 °C and relative humidity ≥ 95%). The final appearance of the prepared specimens is shown in Figure 4.

### 2.2. Test Method

The compressive strength (*f*_c_) and elastic modulus (*E*) were tested according to Chinese National Standard GB/T 50081-2019 [42]. For each mix proportion, three 150 mm cubes and six Φ150 × 300 mm cylinders were cast and standard-cured. The cubic compressive strength was directly obtained from cube tests. The elastic modulus was determined using cylinder specimens under uniaxial compression, calculated as the slope of the secant on the stress–strain curve between 0.5 MPa and one-third of the uniaxial compressive strength.

**Figure 5:**
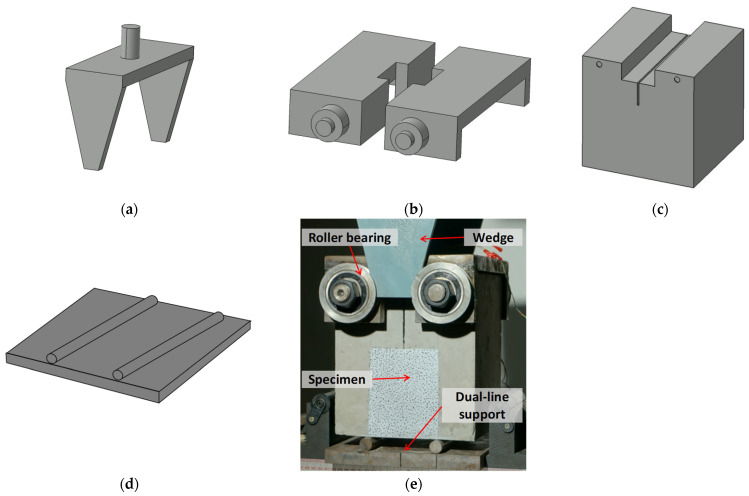
Testing arrangement for the wedge-splitting test [15]: (**a**) wedge-shaped loading device; (**b**) load-transfer device equipped with roller bearings; (**c**) specimen; (**d**) dual-line support system; (**e**) completed test setup.

The wedge-splitting test (WST) specimen offers the advantage of minimizing the adverse impact of the specimen’s self-weight on the measured fracture toughness values. Furthermore, the fracture surface of WST specimens is sufficiently large, with depth and width being 7.5 and 12.5 times the maximum coarse aggregate size in the present work, respectively. Therefore, the WST method was selected in this study to determine the double-*K* fracture parameters of concrete. The loading setup for WST specimens is illustrated in Figure 5. The top part consists of a wedge-shaped loading device (Figure 5a) designed to transfer the load from the upper compression platen. The load is transmitted via two identical wedge-shaped steel plates to a load-transfer device equipped with roller bearings (Figure 5b). The inner side of the load-transfer device is in tight contact with the pre-formed groove of the specimen, ensuring the load is ultimately applied to the specimen (Figure 5c). To maintain specimen stability during testing, a dual-line support system was employed (Figure 5d), where two round steel bars were placed at the quarter points along the specimen bottom. One bar was fixed, while the other was free to roll. A photograph of the completed test setup is shown in Figure 5e. A constant *CMOD*-controlled loading rate of 0.1 mm/min was maintained for the wedge-splitting tests. Both the basic mechanical and fracture tests were conducted using a DNS100 electronic universal testing machine, manufactured by China Testing Equipment Co., Ltd., Changchun, Jilin Province, China.

### 2.3. Determination of Double-K Fracture Toughness

#### 2.3.1. Unstable Toughness

The unstable toughness ($K_{\mathrm{IC}}^{\mathrm{un}}$, MPa·m^1/2^) for the WST specimen is given by the following expression, which relates the peak horizontal load (*P*_h,max_, N) to the critical effective crack length (*a*_c_, m) [13]:

(1) $$K_{\mathrm{IC}}^{\mathrm{un}}=\frac{P_{h,\max}}{B\sqrt{D}}F(\alpha_{c})$$

(2) $$\alpha_{c}=\frac{a_{c}}{D}$$

(3) $$F(\alpha_{c})=3.675[1-0.12(\alpha_{c}-0.45)]{\left(1-\alpha_{c}\right)}^{-3/2}$$

(4) $$a_{c}=D[1-\sqrt{13.18/(\frac{CMOD_{c}\cdot E\cdot B}{P_{h,\max}}+9.16})]$$

where *D* and *B* are the effective depth and thickness of the specimen (m), respectively; *CMOD*_C_ denotes the critical crack mouth opening displacement (μm); and *E* is the elastic modulus (MPa). Additionally, it should be noted that considering a wedge angle *θ* of 15°, the relationship *P*_h,max_ = 1.866 *P*_max_ (N) applies.

#### 2.3.2. Initiation Toughness

Upon the initiation of a crack in concrete subjected to load, a remarkable phenomenon unfolds as stable propagation ensues. This process leads to an elevation in the crack-tip stress intensity factor, progressing from $K_{\mathrm{IC}}^{\mathrm{ini}}$ to $K_{\mathrm{IC}}^{\mathrm{un}}$. This increase, driven by the cohesive forces within the fictitious crack zone, is referred to as cohesive fracture toughness, denoted by $K_{\mathrm{IC}}^{c}$ (MPa·m^1/2^). It is a critical measure, elegantly captured by the following equation:

(5) $$K_{\mathrm{IC}}^{c}=K_{\mathrm{IC}}^{\mathrm{un}}-K_{\mathrm{IC}}^{\mathrm{ini}}$$

The determination of the cohesive fracture toughness $K_{\mathrm{IC}}^{c}$ primarily involves numerical methods [12,43] and weight function approaches [19,20]. The numerical method involves a relatively complex solving process and requires some specialized techniques for implementation. In contrast, the weight function method is more straightforward and demonstrates high accuracy. Based on the universal weight function expression, Kumar and Barai [19] developed a five-term weight function expression for $K_{\mathrm{IC}}^{c}$.

(6) $$\begin{array}{l} K_{\mathrm{IC}}^{c}=\frac{2}{\sqrt{2\pi a_{c}}}\left\{A_{1}a_{c}\left[2s^{1/2}+M_{1}s+\frac{2}{3}M_{2}s^{3/2}+\frac{1}{2}M_{3}s^{2}+\frac{2}{5}M_{4}s^{5/2}\right]\right.\\ \left.A_{2}{a_{c}}^{2}\left[\frac{4}{3}s^{3/2}+\frac{1}{2}M_{1}s^{2}+\frac{4}{15}M_{2}s^{5/2}+\frac{1}{6}M_{3}\{1-{\left(a_{0}/a_{c}\right)}^{3}-3sa_{0}/a_{c}\}+\frac{4}{35}M_{4}s^{7/2}\right]\right\} \end{array}$$

*A*_1_ = *σ*_s_(*CTOD*_c_) (7)

*A*_2_ = (*f*_t_ − *A*_1_)/(*a*_c_ − *a*_0_) (8)

*s* = 1 − *a*_0_/*a*_c_ (9)

where *M*_1_, *M*_2_, *M*_3_, and *M*_4_ are weight function parameters that can be expressed as polynomials of *a*_c_/D, as detailed in the research by Kumar and Barai [19]; *a*_0_ represents the initial notch length (m); *a*_c_ denotes the critical effective crack length of the specimen (m); additionally, *A*_1_ denotes the critical cohesive stress at the initial notch tip (MPa), i.e., *σ*_s_(*CTOD*_c_), which is determined using the nonlinear softening model proposed by Reinhardt et al. [44].

(10) $$\sigma_{s}(CTOD_{C})=f_{t}\left\{\left[1+{\left(\frac{c_{1}CTOD_{C}}{w_{c}}\right)}^{3}\right]\exp\left(-\frac{c_{2}CTOD_{C}}{w_{c}}\right)-\frac{CTOD_{C}}{w_{c}}(1+{c_{1}}^{3})\exp(-c_{2})\right\}$$

where *f*_t_ is the tensile strength (MPa); *CTOD*_c_ represents the critical crack tip opening displacement (μm); *c*_1_, *c*_2_, and *w*_c_ can be determined using the following expressions [45]:

(11) $$c_{1}={\left(d_{\max}/8\right)}^{0.75}$$

(12) $$c_{2}=(0.92-d_{\max}/400)\lambda$$

(13) $$w_{c}=\alpha_{F}G_{\mathrm{IF}}/f_{\mathrm{tm}}$$

(14) $$\lambda =10-{\left[f_{\mathrm{ck}}/(2f_{\mathrm{ck}0})\right]}^{0.7}$$

(15) $$\alpha_{F}=\lambda -{d_{\max}}^{0.9}/8$$

(16) $$f_{\mathrm{ck}}=f_{\mathrm{cm}}-8$$

where *d*_max_ is the maximum coarse aggregate size (mm); *f*_cm_ and *f*_tm_ are the mean compressive strength and tensile strength (MPa), respectively; *f*_ck_ denotes the characteristic concrete strength, which can be taken as *f*_ck_ = *f*_cm_ − 8 MPa, and *f*_ck0_ = 10 MPa.

#### 2.3.3. Fracture Energy

Fracture energy *G*_IF_ (N/m) is defined as the energy dissipated per unit fracture area. RILEM 50-FMC [46] provides the following expression for determining the Mode I fracture energy using the work-of-fracture method:

(17) $$G_{\mathrm{IF}}=\frac{W_{F}}{(D-a_{0})B}$$

where *W*_F_ (J) represents the work performed by the horizontal load *P*_h_ during the entire testing process, calculated as the area under the curve plotting load against the crack mouth opening displacement; *D*, *B*, and *a*_0_ denote the effective depth, thickness, and initial notch length of the specimen (m), respectively.

#### 2.3.4. Characteristic Length

Hillerborg et al. [8] demonstrated that *G*_IF_ cannot adequately characterize the brittleness or ductility of materials. To address this limitation, they proposed the characteristic length *l*_ch_ (mm) as a more effective parameter for this purpose.

(18) $$l_{\mathrm{ch}}=\frac{EG_{\mathrm{IF}}}{{f_{t}}^{2}}$$

where *E* (GPa) and *f*_t_ (MPa) represent the elastic modulus and tensile strength of concrete, respectively. A larger value of *l*_ch_ indicates greater concrete ductility and enhanced resistance to crack propagation.

## 3. Results and Discussion

### 3.1. Fracture Behaviors

**Figure 6:**
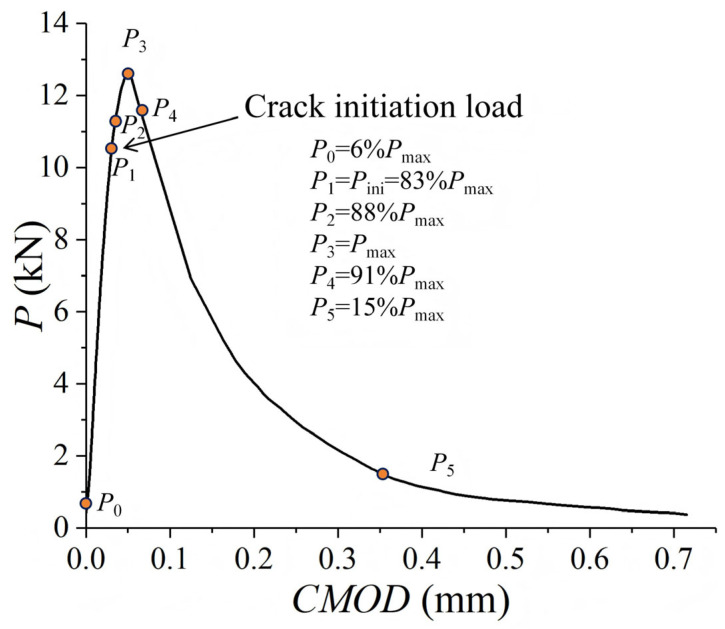
*P-CMOD* curve for specimen WDO4.

The Mode I fracture process of concrete, as explained by the double-*K* fracture theory, occurs in three distinct stages: crack initiation, stable propagation, and unstable fracture. This progression is illustrated in the typical load versus crack mouth opening displacement (*P*-*CMOD*) curve, as shown in Figure 6. The transition between these stages is marked by the initial cracking load *P*_ini_ and the peak load *P*_max_ (unstable load).

- Stage I: From the start of loading to the initial cracking load P_ini_

During this stage, the relationship between load and *CMOD* is approximately linear. The specimen remains in a nearly linear elastic state, with no visible macroscopic cracks observed in the fracture ligament.

- Stage II: From P_ini_ to P_max_, representing the stable crack propagation

**Figure 7:**
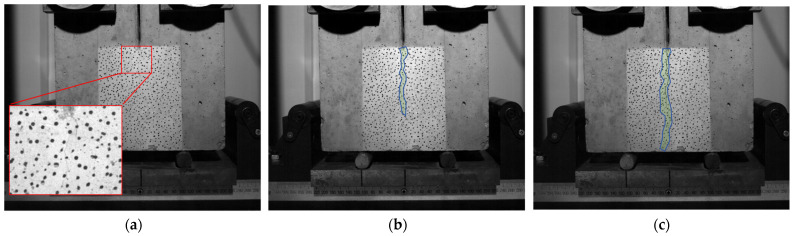
The fracture process for specimen WD04 of (**a**) at *P*_1_, (**b**) at *P*_4_, and (**c**) at *P*_5_.

As the load increases, randomly distributed microcracks gradually coalesce into a microcrack band, eventually forming a dominant macroscopic crack (Figure 7a). This process can be regarded as a phenomenon of damage localization. The initial cracking load *P*_ini_ denotes the appearance of a macrocrack. With further loading, the dominant crack propagates stably, and the *P-CMOD* curve exhibits significant nonlinearity.

- Stage III: Post-peak softening stage, representing the unstable fracture

**Figure 8:**
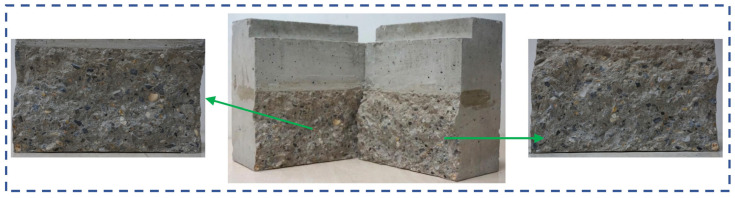
The fracture surface for specimen WC01 [15].

After reaching *P*_max_, the crack enters the stage of unstable propagation. The load decreases while the crack rapidly extends and traverses the entire fracture ligament (Figure 7b). Ultimately, the specimen completely fractures along the dominant crack (Figure 7c), resulting in two relatively intact blocks. No significant secondary cracks are observed on the fracture surface (Figure 8).

**Figure 9:**
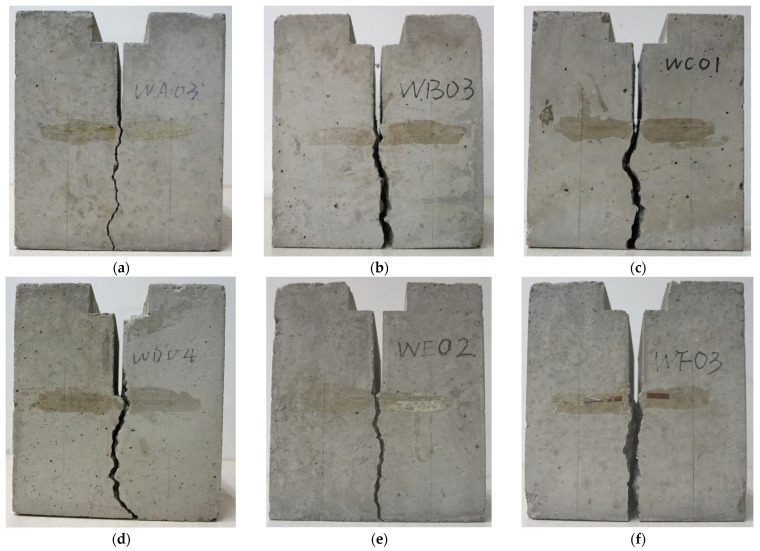
The fracture patterns for specimens numbered (**a**) WA03, (**b**) WB03, (**c**) WC01, (**d**) WD04, (**e**) WE02, and (**f**) WF03 [15].

Typical fracture patterns under different coarse aggregate volume fractions were selected and are presented in Figure 9. All specimens generally exhibit a similar pattern: the macroscopic dominant crack initiates at the tip of the prefabricated notch, traverses the fracture ligament, and ultimately leads to the complete separation of the specimen along the fracture plane. Meanwhile, the cracking trajectories show significant tortuosity, which is attributed to the heterogeneity of concrete. The obstruction effect of high-strength phases (e.g., coarse aggregates) causes the cracks to deflect.

Furthermore, a non-monotonic trend is also observed for crack path tortuosity: it intensifies with the coarse aggregate volume fraction up to 37% (Figure 9a–c), peaking at this point, and subsequently diminishes as the fraction reaches 50% (Figure 9d–f). This suggests potential changes in the homogeneity and brittleness of the concrete material. In subsequent sections, a detailed discussion will be provided from the perspective of energy dissipation, combined with fracture parameters.

### 3.2. Mechanical and Fracture Parameters

#### 3.2.1. Compressive Strength and Elastic Modulus

The average values of basic mechanical and fracture parameters for concrete specimens with each mix proportion are listed in Table 4.

**Figure 10:**
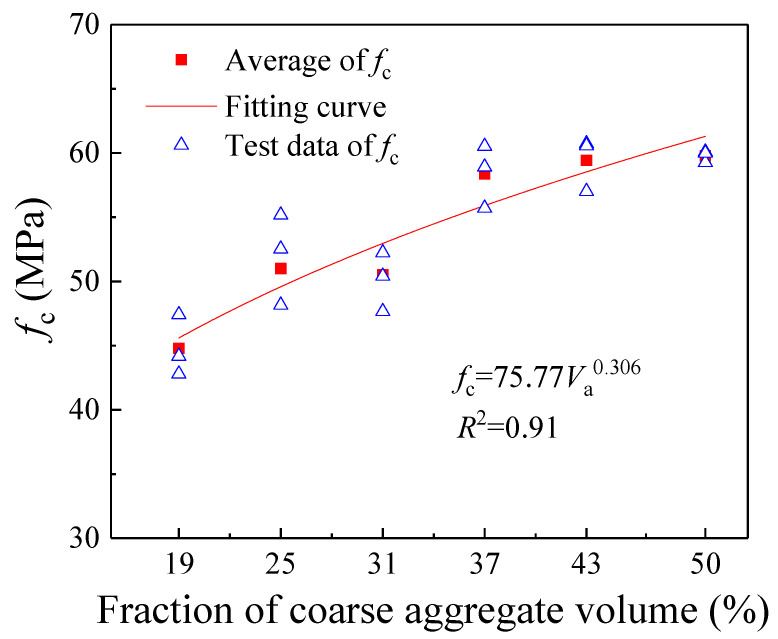
The change in compressive strength with different coarse aggregate volume fractions.

**Figure 11:**
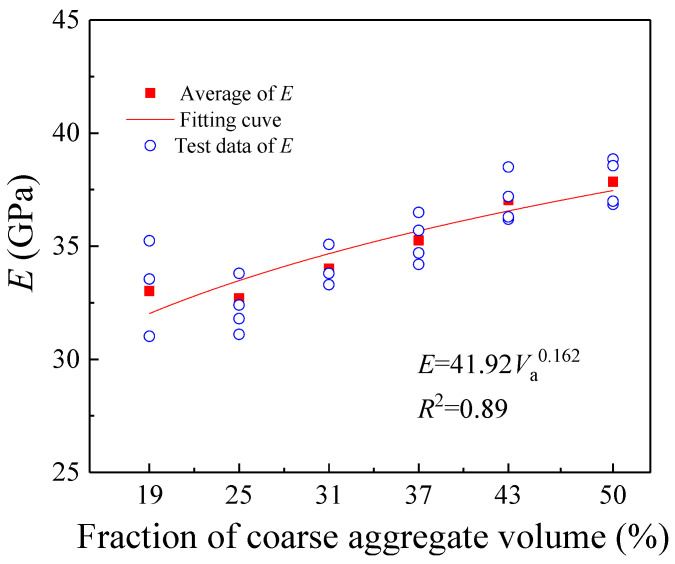
The change in elastic modulus with different coarse aggregate volume fractions.

Figure 10 and Figure 11 present the variations in concrete compressive strength (*f*_c_) and elastic modulus (*E*) with increasing coarse aggregate volume fraction (*V*_a_). As *V*_a_ rises from 19% to 50%, both *f*_c_ and *E* exhibit a gradual increase. Furthermore, as illustrated in Figure 10, when *V*_a_ increases from 19% to 37%, the compressive strength *f*_c_ experiences a significant growth of 22.12%. This increase is substantially higher than the 2.43% rise observed when *V*_a_ further increases from 37% to 50%. Therefore, the reinforcing effect of coarse aggregates is more pronounced at lower volume fractions (19–37%), while its efficacy relatively diminishes when the volume fraction becomes excessively high (43–50%). The primary reason for this lies in the reduced proportion of fine aggregates (sand) and the increased cement content in the mortar matrix as the coarse aggregate volume fraction rises. These changes improve the strength of the mortar matrix, narrowing the strength gap between the mortar and the coarse aggregates. In certain regions, the mortar may even exhibit higher strength than the coarse aggregates, thereby substantially diminishing the reinforcing effect of the coarse aggregates.

From a mesoscopic perspective, the coarse aggregate volume fraction (*V*_a_) is intrinsically linked to the roughness (i.e., compactness) of the concrete’s internal structure and the thickness of the paste layer between adjacent coarse aggregates. Based on morphological theory, as *V*_a_ increases, the roughness of the internal structure increases, resulting in denser concrete with higher strength [47].

In summary, the results of the aforementioned theoretical analysis are consistent with the trends observed in the present experiments. Furthermore, regression analysis of the measured experimental data revealed the following quantitative relationships between the compressive strength (*f*_c_), elastic modulus (*E*), and the coarse aggregate volume fraction (*V*_a_):

(19) $$f_{c}=75.77{V_{a}}^{0.306}(\mathrm{MPa})\;R^{2}=0.91$$

(20) $$E=41.92{V_{a}}^{0.162}(\mathrm{GPa})\;R^{2}=0.89$$

#### 3.2.2. Tensile Strength and Fracture Parameters

**Figure 12:**
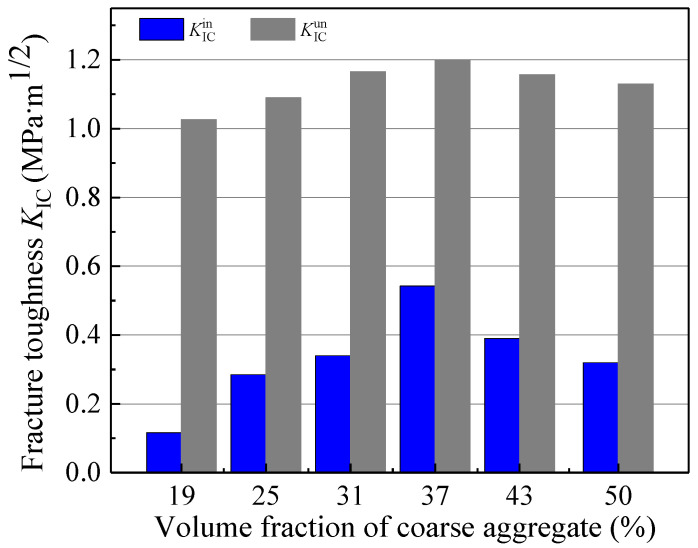
The change in double-*K* fracture parameters with different coarse aggregate volume fractions.

**Figure 13:**
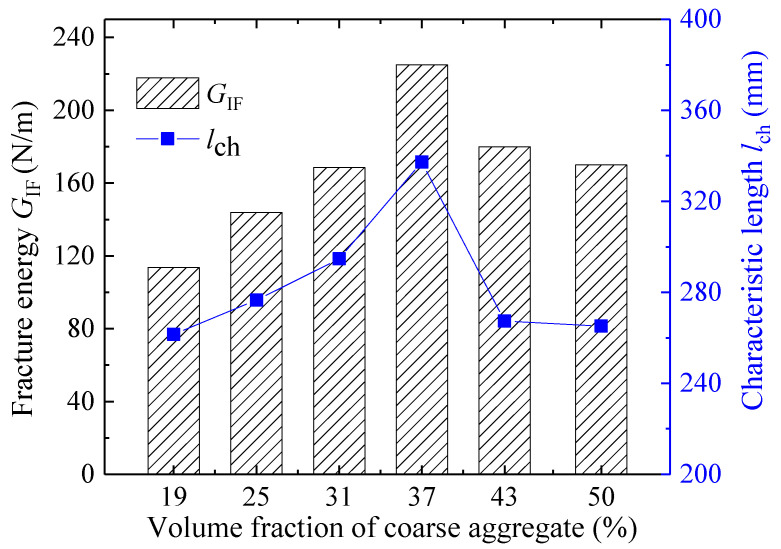
The change in fracture energy and characteristic length with different coarse aggregate volume fractions.

As shown in Figure 12 and Figure 13, the initiation toughness $K_{\mathrm{IC}}^{\mathrm{ini}}$, unstable toughness $K_{\mathrm{IC}}^{\mathrm{un}}$, and fracture energy *G*_IF_ of concrete exhibit a trend of initial increase followed by a decrease with rising coarse aggregate volume fraction (*V*_a_), reaching their peak values at a volume fraction of 37%. These results indicate the existence of an optimal range for the aforementioned fracture parameters, which provides valuable guidance for concrete mix design.

**Figure 14:**
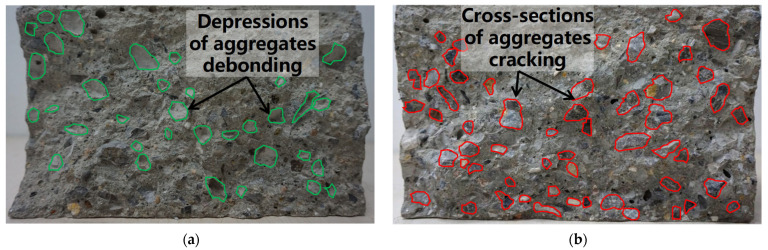
The fracture surfaces and crack paths for specimens numbered (**a**) WA03 and (**b**) WE02.

The observed trend is a result of how the coarse aggregate volume fraction influences the geometric characteristics of the crack path. It should be noted that in the concrete mix design employed in this study, the total weight of coarse and fine aggregates remains constant across different *V*_a_. Consequently, as *V*_a_ increases, the corresponding volume fraction and weight of fine aggregates (river sand) decrease proportionally. At lower *V*_a_, the higher weight ratio of river sand in the mortar matrix yields comparatively weaker mortar and ITZ. Therefore, cracks primarily propagate around the coarse aggregates through the interfacial transition zone (ITZ) and mortar matrix, with coarse aggregate debonding resulting in rougher fracture surfaces (Figure 14a). As *V*_a_ increases to 37%, the obstruction effect of coarse aggregates on crack propagation becomes more pronounced, leading to more tortuous crack paths, increased fracture surface roughness, and larger fracture areas, thereby enhancing energy dissipation and improving fracture parameters.

At higher coarse aggregate volume fractions (37% to 50%), the smaller proportion of river sand in the mortar leads to significant enhancements in the mechanical properties of the mortar matrix. Consequently, when *V*_a_ further increases to 50%, the higher strength of the mortar matrix causes cracks to preferentially directly propagate through the coarse aggregates. This results in shorter crack paths and smoother fracture surfaces (Figure 14b), consequently reducing energy dissipation. Additionally, the higher coarse aggregate content reduces inter-aggregate spacing, impairing the paste coating capacity. Furthermore, as the coarse aggregate volume fraction increases, the total surface area of coarse aggregates increases, leading to a larger overall area of the surrounding ITZs, which are known to be a weaker phase in concrete. These factors collectively degrade the fracture performance of concrete.

Furthermore, as presented in Table 4 and Figure 12, when *V*_a_ increased from 19% to 37%, $K_{\mathrm{IC}}^{\mathrm{ini}}$ and $K_{\mathrm{IC}}^{\mathrm{un}}$ increased by 350% and 16.5%, respectively. In contrast, when the volume fraction further increased from 37% to 50%, they decreased by 40.7% and 5.8%, respectively. These results clearly demonstrate that the coarse aggregate volume fraction exerts a more pronounced influence on $K_{\mathrm{IC}}^{\mathrm{ini}}$ than $K_{\mathrm{IC}}^{\mathrm{un}}$.

**Figure 15:**
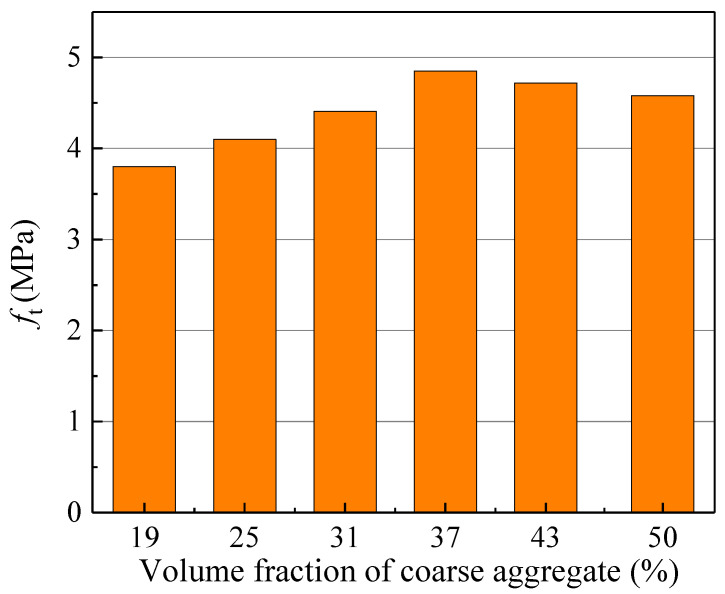
The change in tensile strength with different coarse aggregate volume fractions.

**Figure 16:**
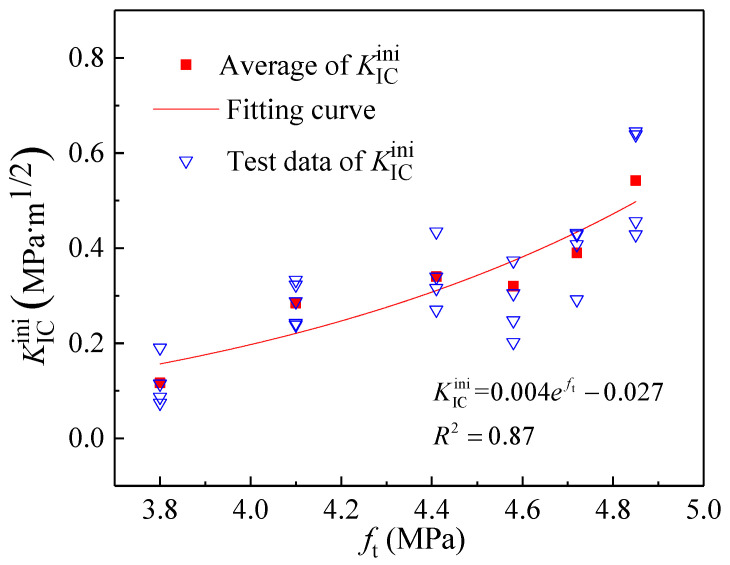
The change in initiation toughness ($K_{\mathrm{IC}}^{\mathrm{ini}}$) with different tensile strengths (*f*_t_).

**Figure 17:**
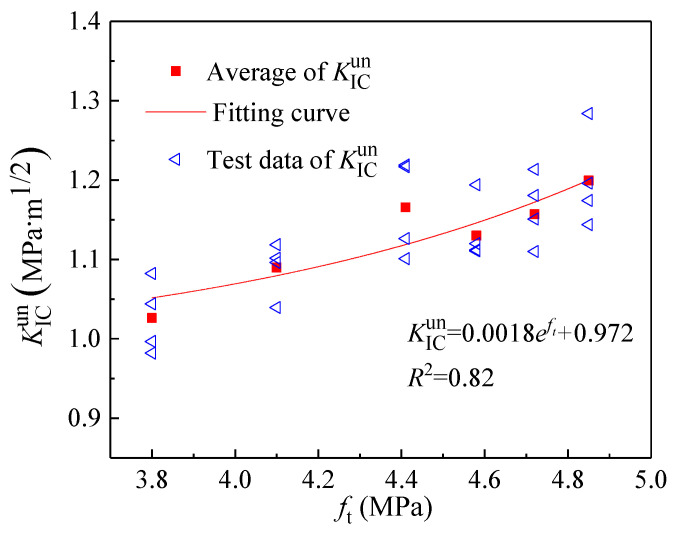
The change in unstable toughness ($K_{\mathrm{IC}}^{\mathrm{un}}$) with different tensile strengths (*f*_t_).

**Figure 18:**
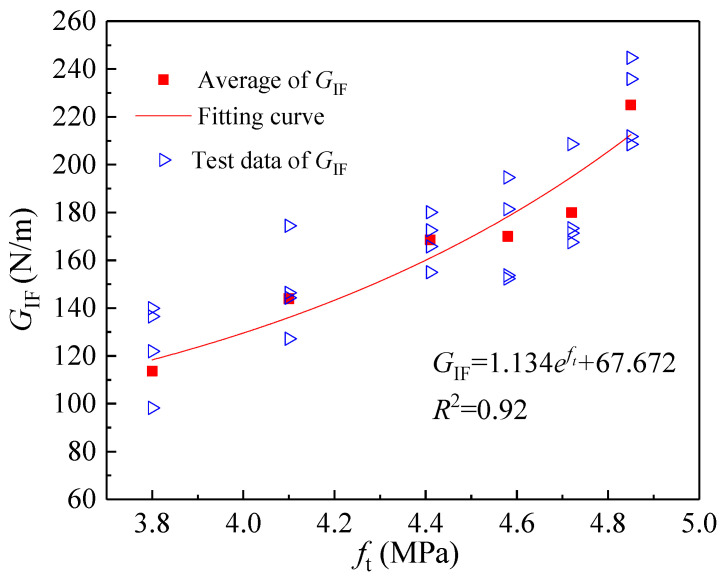
The change in fracture energy (*G*_IF_) with different tensile strengths (*f*_t_).

As the simplest and most direct indicator of concrete’s resistance to tensile deformation, the tensile strength *f*_t_ exhibits a variation trend with coarse aggregate volume fraction consistent with that of the double-*K* fracture toughness and fracture energy (Figure 15). Consequently, *f*_t_ can be considered for calculating the initiation toughness $K_{\mathrm{IC}}^{\mathrm{ini}}$, unstable toughness $K_{\mathrm{IC}}^{\mathrm{un}}$, and fracture energy *G*_IF_. Figure 16, Figure 17 and Figure 18 present the variations in $K_{\mathrm{IC}}^{\mathrm{ini}}$, $K_{\mathrm{IC}}^{\mathrm{un}}$, and *G*_IF_ with *f*_t_, respectively. All three parameters demonstrate significant increases with rising *f*_t_. Regression analysis of the measured experimental data reveals that $K_{\mathrm{IC}}^{\mathrm{ini}}$, $K_{\mathrm{IC}}^{\mathrm{un}}$, and *G*_IF_ exhibit exponential relationships with *f*_t_. The choice of the exponential functional form was motivated by its successful application in modeling concrete fracture properties in previous studies [48].

(21) $$K_{\mathrm{IC}}^{\mathrm{ini}}=0.004e^{f_{t}}-0.027\;(\mathrm{MPa}\cdot m^{1/2})\;R^{2}=0.87$$

(22) $$K_{\mathrm{IC}}^{\mathrm{un}}=0.0018e^{f_{t}}+0.972\;(\mathrm{MPa}\cdot m^{1/2})\;R^{2}=0.82$$

(23) $$G_{\mathrm{IF}}=1.134e^{f_{t}}+97.672\;(N/m)\;R^{2}=0.92$$

#### 3.2.3. Safety Warning Parameter from the Double-*K* Fracture Toughness

The criterion of stable crack propagation, $K_{\mathrm{IC}}^{\mathrm{ini}}$ < *K* < $K_{\mathrm{IC}}^{\mathrm{un}}$, established by Xu et al. [12,13], has significant implications for the integrity assessment of critical engineering structures and other large-scale concrete projects, as it can serve as an early warning before catastrophic failure. However, the current analysis remains qualitative and cannot yet provide quantitative safety predictions prior to an unstable fracture occurring.

The evolution of the stress intensity factor (*K*_IC_) throughout the entire stable crack propagation, governed by the cohesive fracture toughness $K_{\mathrm{IC}}^{c}$, measures the progression of *K*_IC_ from $K_{\mathrm{IC}}^{\mathrm{ini}}$ to $K_{\mathrm{IC}}^{\mathrm{un}}$. Consequently, the normalized rate of increase in *K*_IC_ from crack initiation to unstable propagation can be described by the ratio of $K_{\mathrm{IC}}^{c}$ to $K_{\mathrm{IC}}^{\mathrm{un}}$. The expression is given as follows:

(24) $$\delta =\frac{K_{\mathrm{IC}}^{\mathrm{un}}-K_{\mathrm{IC}}^{\mathrm{ini}}}{K_{\mathrm{IC}}^{\mathrm{un}}}=\frac{K_{\mathrm{IC}}^{c}}{K_{\mathrm{IC}}^{\mathrm{un}}}$$

In this equation, *δ* represents the pre-peak ductility index of concrete, which can serve as a safety warning parameter prior to unstable fracture of concrete structures. Its value ranges between 0 and 1 (0 < *δ* < 1). A value of *δ* closer to 1 indicates slower unstable crack propagation. When *δ* = 0, $K_{\mathrm{IC}}^{\mathrm{ini}}$ = $K_{\mathrm{IC}}^{\mathrm{un}}$, meaning the material exhibits no stable propagation stage and undergoes standard brittle fracture—a scenario that is impossible for quasi-brittle materials like concrete.

**Figure 19:**
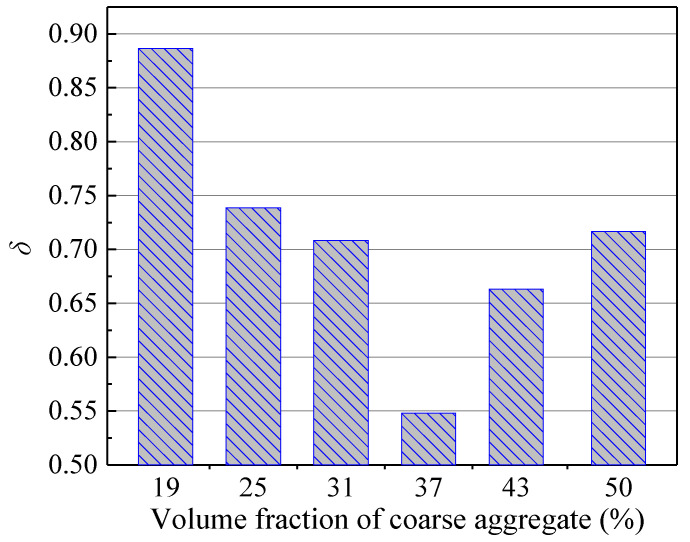
The change in safety warning parameter (*δ*) with different coarse aggregate volume fractions.

Therefore, the mix design of critical concrete structures should adhere to the following principles: First, $K_{\mathrm{IC}}^{\mathrm{ini}}$ must be sufficiently high to prevent cracking under normal service conditions. Second, an adequate value of *δ* should be ensured to provide more time for personnel evacuation and emergency repairs. The variation in *δ* with coarse aggregate volume fraction (*V*_a_) is shown in Figure 19. As *V*_a_ increases from 19% to 50%, *δ* initially decreases and then increases, reaching its minimum at 37%. This trend is opposite to that observed for the initiation toughness $K_{\mathrm{IC}}^{\mathrm{ini}}$ under varying *V*_a_. Consequently, the selection of coarse aggregate volume fraction for critical concrete structures requires a comprehensive evaluation. When the volume fraction ranges between 25% and 31%, both *δ* and $K_{\mathrm{IC}}^{\mathrm{ini}}$ attain relatively high values, while the consumption of coarse aggregates remains moderate. This range can thus serve as a practical reference for concrete structure design. Furthermore, while the characteristic length *l*_ch_ represents the ductility of concrete throughout the entire hardening–softening process, *δ* can be regarded as a ductility indicator for the pre-peak nonlinear stage.

### 3.3. Directions for Future Work

Future work should extend beyond volume fraction to examine the synergistic effects of other aggregate properties, such as maximum size, shape (e.g., rounded vs. angular), surface texture, and mineralogical type, on the double-*K* fracture parameters and the safety warning parameter (*δ*).

## 4. Conclusions

From the experimental and analytical investigations undertaken in this work, the following conclusions can be drawn:

- Fracture Process and Crack Path: The fracture process of the wedge-splitting test (WST) specimen, characterized by the *P-CMOD* curve, consistently exhibits three distinct stages: nearly linear elastic state, nonlinear stable crack propagation, and post-peak unstable fracture. The tortuosity of the macroscopic crack path initially increases with the coarse aggregate volume fraction (*V*_a_) up to 37%, indicating enhanced crack deflection and energy dissipation, but diminishes thereafter as *V*_a_ increases to 50%, suggesting a transition in cracking behavior.
- Influence on Basic Mechanical Properties: Both the compressive strength (*f*_c_) and elastic modulus (*E*) monotonically increase with the increase in *V*_a_ from 19% to 50%. However, the reinforcing effect of coarse aggregates is more pronounced in the lower *V*_a_ (19–37%), with a significant 22.12% increase in *f*_c_, compared to a marginal 2.43% increase in the higher range (37–50%).
- Optimal Fracture Performance: The tensile strength (*f*_t_), double-*K* fracture toughness (initiation toughness $K_{\mathrm{IC}}^{\mathrm{ini}}$ and unstable toughness $K_{\mathrm{IC}}^{\mathrm{un}}$), and fracture energy (*G*_IF_) demonstrate a non-monotonic relationship with *V*_a_, peaking at a *V*_a_ of 37%. This indicates the existence of an optimal *V*_a_ for maximizing fracture resistance. Notably, $K_{\mathrm{IC}}^{\mathrm{ini}}$ is significantly more sensitive to changes in *V*_a_ than $K_{\mathrm{IC}}^{\mathrm{un}}$, evidenced by a 350% increase from *V*_a_ = 19% to 37%, compared to a 16.5% increase for $K_{\mathrm{IC}}^{\mathrm{un}}$.
- Quantitative Relationships: Strong exponential correlations were established between *f*_t_ and $K_{\mathrm{IC}}^{\mathrm{ini}}$, $K_{\mathrm{IC}}^{\mathrm{un}}$, and *G*_IF_. These relationships facilitate the prediction of fracture properties based on the more readily measurable tensile strength.
- Safety Warning Parameter: A novel safety warning parameter (*δ*), defined as the ratio of cohesive toughness to unstable toughness, was proposed to quantitatively assess the pre-peak ductility and provide a warning margin before unstable fracture. For critical concrete structures, a range of *V*_a_ (25–31%) is recommended, as it offers a balanced combination of high crack initiation resistance and adequate safety warning capacity for critical engineering structures.

In summary, this study underscores the critical role of coarse aggregate volume fraction in governing the fracture behavior of concrete and provides quantitative insights for optimizing mix design in engineering applications demanding high fracture resistance and structural safety.

## Figures and Tables

**Table 1:** Composition of cement [15,35].

Chemical Composition wt (%)							
SiO_2_	Fe_2_O_3_	Al_2_O_3_	CaO	MgO	Na_2_O	K_2_O	SO_3_
22.27	2.95	6.37	60.23	4.52	0.13	0.52	2.51
**Mineral Composition wt (%)**							
3CaO·Al_2_O_3_		3CaO·SiO_2_		2CaO·SiO_2_		4CaO·Al_2_O_3_·Fe_2_O_3_	
6.9		49.58		28.13		8.62	

**Table 2:** The physical properties of cement [15,35].

Specific Surface Area (m^2^/kg)	*f*_c_ (MPa)		*f*_t_ (MPa)		Setting Time (min)	
329	3 d	28 d	3 d	28 d	initial	final
	23.5	50.3	5.6	8.8	196	258

**Table 3:** Compositions of concretes [15,35].

Group	*V*_a_ ^1^ (%)	Specimens	Unit Mass (kg/m^3^)					
			Cement	Sand	Coarse Aggregate	Limestone Powder	Water	Superplasticizer
A	19	WA01~WA05	490	1167	500	96	179.3	5
B	25	WB01~WB05	490	1000	667	96	179.3	5.5
C	31	WC01~WC05	490	834	833	96	179.3	6.1
D	37	WD01~WD05	490	667	1000	96	179.3	7.4
E	43	WE01~WE05	490	500	1167	96	179.3	7.9
F	50	WF01~WF05	490	334	1333	96	179.3	8.4

^1^ *V*_a_ is the coarse aggregate volume fraction.

**Table 4:** Mechanical and fracture parameters of concrete by experimental tests.

*V*_a_ (%)	*f*_c_ (MPa)	*f*_t_ (MPa)	*E* (GPa)	$K_{\mathrm{IC}}^{\mathrm{ini}}$ (MPa·m^1/2^)	$K_{\mathrm{IC}}^{\mathrm{un}}$ (MPa·m^1/2^)	*G*_IF_ (N/m)	*l*_ch_ (mm)
19	44.79	3.8	33.23	0.12	1.01	113.66	261.57
25	51.96	4.1	32.3	0.28	1.09	143.94	276.58
31	50.08	4.41	34.01	0.34	1.17	168.56	294.78
37	58.36	4.85	35.26	0.54	1.20	225.01	337.28
43	59.43	4.72	37.04	0.39	1.16	180.23	267.41
50	59.78	4.58	37.85	0.32	1.13	170.52	265.19

## Data Availability

The original contributions presented in this study are included in the article. Further inquiries can be directed to the corresponding author.

## References

[1] Mukhtar F., El-Tohfa A. (2023). A Review On Fracture Propagation in Concrete: Models, Methods, and Benchmark Tests. Eng. Fract. Mech..

[2] Tang Y., Xiao J., Zhang H., Wang D., Zhang M., Zhang J. (2024). Effect of Accelerated Carbonation of Fully Recycled Aggregates On Fracture Behaviour of Concrete. Cem. Concr. Compos..

[3] Du J., Wang J., Zhu F. (2024). Influence of Interaction Between Microcracks and Macrocracks On Crack Propagation of Asphalt Concrete. Materials.

[4] Chen H., Zhuo Y., Li D., Huang Y. (2024). Fracture Toughness of Ordinary Plain Concrete Under Three-Point Bending Based On Double-K and Boundary Effect Fracture Models. Materials.

[5] Yu K., Qing L., Hu Y. (2025). The Effects of Specimen Size and Aggregate On the Evolution of the Fracture Process Zone in Concrete: A Mesoscale Investigation. Compos. Struct..

[6] Shah S.P., McGarry F.J. (1971). Griffith Fracture Criterion and Concrete. J. Eng. Mech. Div..

[7] Elices M., Planas J. (1996). Fracture Mechanics Parameters of Concrete. Adv. Cem. Based Mater..

[8] Hillerborg A., Modéer M., Petersson P.E. (1976). Analysis of Crack Formation and Crack Growth in Concrete by Means of Fracture Mechanics and Finite Elements. Cem. Concr. Res..

[9] Bažant Z.P., Oh B.H. (1983). Crack Band Theory for Fracture of Concrete. Matériaux Constr..

[10] Jenq Y.S., Shah S.P. (1985). Two Parameter Fracture Model for Concrete. J. Eng. Mech..

[11] Bažant Z.P. (1984). Size Effect in Blunt Fracture: Concrete, Rock, Metal. J. Eng. Mech..

[12] Xu S., Reinhardt H.W. (1999). Determination of Double-K Criterion for Crack Propagation in Quasi-Brittle Fracture, Part II: Analytical Evaluating and Practical Measuring Methods for Three-Point Bending Notched Beams. Int. J. Fract..

[13] Xu S., Reinhardt H.W. (1999). Determination of Double-K Criterion for Crack Propagation in Quasi-Brittle Fracture, Part III: Compact Tension Specimens and Wedge Splitting Specimens. Int. J. Fract..

[14] Xu S., Reinhardt H.W. (1999). Determination of Double-Determination of Double-K Criterion for Crack Propagation in Quasi-Brittle Fracture Part I: Experimental Investigation of Crack Propagation. Int. J. Fract..

[15] Chen Y., Feng J. (2022). Experimental Study On Effect of Coarse Aggregate Volume Fraction On Mode I and Mode II Fracture Behavior of Concrete. J. Adv. Concr. Technol..

[16] Wei X., Chen H., Tang Y., Li H. (2025). Simplified Determination of Double-K Fracture Parameters of Nuclear Graphite Using the Peak Load. Theor. Appl. Fract. Mech..

[17] Zhang P., Yang Y., Wang J., Jiao M., Ling Y. (2020). Fracture Models and Effect of Fibers On Fracture Properties of Cementitious Composites—A Review. Materials.

[18] Qing L., Li Q. (2013). A Theoretical Method for Determining Initiation Toughness Based On Experimental Peak Load. Eng. Fract. Mech..

[19] Kumar S., Barai S.V. (2009). Determining Double-K Fracture Parameters of Concrete for Compact Tension and Wedge Splitting Tests Using Weight Function. Eng. Fract. Mech..

[20] Kumar S., Barai S.V. (2008). Influence of Specimen Geometry On Determination of Double-K Fracture Parameters of Concrete: A Comparative Study. Int. J. Fract..

[21] Kucharczyková B., Šimonová H., Kocáb D., Topolář L. (2021). Advanced Evaluation of the Freeze–Thaw Damage of Concrete Based On the Fracture Tests. Materials.

[22] Zhuo K., Liu G., Lan X., Zheng D., Wu S., Wu P., Guo Y., Lin J. (2022). Fracture Behavior of Steel Slag Powder-Cement-Based Concrete with Different Steel-Slag-Powder Replacement Ratios. Materials.

[23] Mu Y., Xia H., Yan Y., Wang Z., Guo R. (2022). Fracture Behavior of Basalt Fiber-Reinforced Airport Pavement Concrete at Different Strain Rates. Materials.

[24] Wang Y., Hu S., He Z. (2019). Mechanical and Fracture Properties of Fly Ash Geopolymer Concrete Addictive with Calcium Aluminate Cement. Materials.

[25] Huang Y., Zheng L., Li P., Wang Q., Zhang Y. (2024). Effects of Mix Components On Fracture Properties of Seawater Volcanic Scoria Aggregate Concrete. Materials.

[26] Bušić R., Gazić G., Guljaš I., Miličević I. (2025). Experimental Determination of Double-K Fracture Parameters for Self-Compacting Concrete with Waste Tire Rubber and Silica Fume. Theor. Appl. Fract. Mech..

[27] (2022). Pebble and Crushed Stone for Construction.

[28] Mihashi H., Nomura N., Niiseki S. (1991). Influence of Aggregate Size On Fracture Process Zone of Concrete Detected with Three Dimensional Acoustic Emission Technique. Cem. Concr. Res..

[29] Wolinski S., Hordijk D.A., Reinhardt H.W., Cornelissen H.A.W. (1987). Influence of Aggregate Size On Fracture Mechanics Parameters of Concrete. Int. J. Cem. Compos. Lightweight Concr..

[30] Golewski G.L. (2024). Effect of Coarse Aggregate Type On the Fracture Toughness of Ordinary Concrete. Infrastructures.

[31] Zhou F.P., Barr B.I.G., Lydon F.D. (1995). Fracture Properties of High Strength Concrete with Varying Silica Fume Content and Aggregates. Cem. Concr. Res..

[32] Alyhya W.S., Abo Dhaheer M.S., Al-Rubaye M.M., Karihaloo B.L. (2016). Influence of Mix Composition and Strength On the Fracture Properties of Self-Compacting Concrete. Constr. Build. Mater..

[33] Beygi M.H.A., Kazemi M.T., Nikbin I.M., Amiri J.V., Rabbanifar S., Rahmani E. (2014). The Influence of Coarse Aggregate Size and Volume On the Fracture Behavior and Brittleness of Self-Compacting Concrete. Cem. Concr. Res..

[34] Chen B., Liu J. (2004). Effect of Aggregate On the Fracture Behavior of High Strength Concrete. Constr. Build. Mater..

[35] Chen Y., Feng J., Li H., Meng Z. (2021). Effect of Coarse Aggregate Volume Fraction On Mode II Fracture Toughness of Concrete. Eng. Fract. Mech..

[36] Chen Y., Li X., Li Z., Yuan Y., Feng J. (2025). Toughening and Toughness Degradation of Concrete Under Varying Volume Fractions of Coarse Aggregate. Theor. Appl. Fract. Mech..

[37] (2018). Methods for Chemical Analysis of Cement.

[38] (2022). X-Ray Powder Diffraction Analysis Method for Determining the Phases in Portland Cement Clinker.

[39] (2008). Testing Method for Specific Surface of Cement-Blaine Method.

[40] (2022). Test Method of Cement Mortar Strength (ISO Method).

[41] (2012). Test Methods for Water Requirement of Normal Consistency, Setting Time and Soundness of the Portland Cement.

[42] (2019). Standard for Test Methods of Concrete Physical and Mechanical Properties.

[43] Daniewicz S.R. (1994). Accurate and Efficient Numerical Integration of Weight Functions Using Gauss-Chebyshev Quadrature. Eng. Fract. Mech..

[44] Reinhardt H.W., Cornelissen H.A.W., Hordijk D.A. (1986). Tensile Tests and Failure Analysis of Concrete. J. Struct. Eng..

[45] Comite E.D.B. (1993). Ceb-Fip Model Code 1990: Design Code.

[46] RILEM Technical Committees (1985). Determination of the Fracture Energy of Mortar and Concrete by Means of Three-Point Bend Tests On Notched Beams. Mater. Struct..

[47] Amparano F.E., Xi Y., Roh Y.S. (2000). Experimental Study On the Effect of Aggregate Content On Fracture Behavior of Concrete. Eng. Fract. Mech..

[48] Kim J.K., Lee C.S., Park C.K., Eo S.H. (1997). The Fracture Characteristics of Crushed Limestone Sand Concrete. Cem. Concr. Res..

